# Electrical properties of lead-free 0.98(Na_0.5_K_0.5_)NbO_3_-0.02Ba(Zr_0.52_Ti_0.48_)O_3 _piezoelectric ceramics by optimizing sintering temperature

**DOI:** 10.1186/1556-276X-7-15

**Published:** 2012-01-05

**Authors:** Seung-Hwan Lee, Sung-Gap Lee, Young-Hie Lee

**Affiliations:** 1 Department of Ceramic Engineering, Engineering Research Institute, Gyeongsang National University, Jinju-Si, 660-701, South Korea; 2 Department of Electronic Materials Engineering, Kwangwoon University, Seoul, 139-701, South Korea

**Keywords:** NKN-BZT, lead-free, sintering temperature, piezoelectric properties

## Abstract

Lead-free 0.98(Na_0.5_K_0.5_)NbO_3_-0.02Ba(Zr_0.52_Ti_0.48_)O_3 _[0.98NKN-0.02BZT] ceramics were fabricated by the conventional mixed oxide method with sintering temperature at 1,080°C to 1,120°C. The results indicate that the sintering temperature obviously influences the structural and electrical properties of the sample. For the 0.98NKN-0.02BZT ceramics sintered at 1,080°C to 1,120°C, the bulk density increased with increasing sintering temperature and showed a maximum value at a sintering temperature of 1,090°C. The dielectric constant, piezoelectric constant [*d*_33_], electromechanical coupling coefficient [*k*_p_], and remnant polarization [*P*_r_] increased with increasing sintering temperature, which might be related to the increase in the relative density. However, the samples would be deteriorated when they are sintered above the optimum temperature. High piezoelectric properties of *d*_33 _= 217 pC/N, *k*_p _= 41%, dielectric constant = 1,951, and ferroelectric properties of *P*_r _= 10.3 μC/cm^2 ^were obtained for the 0.98NKN-0.02BZT ceramics sintered at 1,090°C for 4 h.

## Introduction

Lead-based perovskites have been extensively used in industries as sensors, actuators, and transducers due to their outstanding electrical properties. However, the PbO in these materials presents an environmental problem. The studies are now focused on discovering an alternative for lead-based materials. Potassium sodium niobate ((Na, K)NbO_3_) materials are thought to be one of the candidates as substitute systems [1-3]. When hot-pressed, (Na_0.5_K_0.5_)NbO_3 _[NKN] ceramics have been reported to possess high phase transition temperature [*T*_c_] (approximately 420°C), high remnant polarization [*P*_r_] (approximately 33 μC/cm^2^), large piezoelectric longitudinal response [*d*_33_] (approximately 160 pC/N), and high planar coupling coefficients [*k*_p_] (approximately 45%) [4-6]. However, conventionally sintered NKN ceramics show relatively lower electrical properties (*d*_33 _= 70 pC/N, *k*_p _= 25%) due to the difficulty of getting a high density of pure NKN ceramics [7]. To compensate for these problems, NKN-based ceramics (e.g., solid solutions of NKN-LiNbO_3_, NKN-LiTaO_3_, NKN-LiSbO_3_, NKN-Li(Ta,Sb)O_3_, NKN-BaTiO_3_, NKN-SrTiO_3_, NKN-Ba(Zr,Ti)O_3_, and NKN-CaTiO_3_) have received significant attention largely for two reasons: (1) piezoelectric properties exist over an extensive range of temperature and (2) several possibilities for substitution and additions. Among them, Ba(Zr_0.52_Ti_0.48_)O_3 _[BZT] ceramics possess very strong piezoelectric properties (*d*_33 _is approximately 236 pC/N) and ferroelectric properties (*P*_r _is approximately 13 to 18 μC/cm^2^). BZT has the advantage of exhibiting improved piezoelectric properties. However, it has a low Curie temperature (about 100°C), which limits its practical application as a piezoelectric material. In view of the high Curie temperature of NKN ceramics, the NKN-BZT binary system is of much value as a piezoelectric material [8-12]. In this paper, we have fabricated a 0.98(Na_0.5_K_0.5_)NbO_3_-0.02Ba(Zr_0.52_Ti_0.48_) [0.98NKN-0.02BZT] solid solution by a conventional ceramics technique, and the influence of sintering temperatures on the dielectric and piezoelectric properties of the 0.98NKN-0.02BZT ceramics was investigated.

## Experiments

The chemical molecular formula used in this experiment for the perovskite ceramics with (Na, K, Ba) complex A-sites and (Nb, Zr, Ti) complex B-sites is 0.98(Na_0.5_K_0.5_)NbO_3_-0.02Ba(Zr_0.52_Ti_0.48_) ceramics. For specimens prepared by the conventional mixed oxide method from Na_2_CO_3_, K_2_CO_3_, Nb_2_O_5_, BaCO_3_, ZrO_2_, and TiO_2 _as the staring materials, these powders were separately dried in an oven at 100°C for 4 h. They were ball-milled for 24 h using zirconia balls in alcohol. After drying at 110°C for 24 h, the powders were calcined at 850°C for 5 h. The calcined powders were pressed into disk samples of *φ *= 12 mm. The samples were sintered at 1,080°C to 1,120°C for 4 h. After the samples were polished up to 1.0-mm thick, Ag paste was screen-printed on the surfaces as electrodes and then fired at 400°C for 10 min. We used X-ray diffraction [XRD] and scanning electron microscopy [SEM] to analyze the crystalline and microstructures. The dielectric properties were measured using an LCR meter (PM6306, Fluke, Test Equipment Connection Corporation, Lake Mary, FL, USA). Hysteresis loops of the samples were measured by a Sawyer-Tower circuit. The samples were poled under a DC field of 4 kV/mm for 20 min. The *d*_33 _was measured by a *d*_33 _meter (DT-3300, Channel Products Inc., Chesterland, OH, USA). The *k*_p _was calculated by measuring the antiresonance and resonance frequencies. The relative density of the sintered samples was measured by the Archimedes method.

## Results and discussion

**Figure 1:**
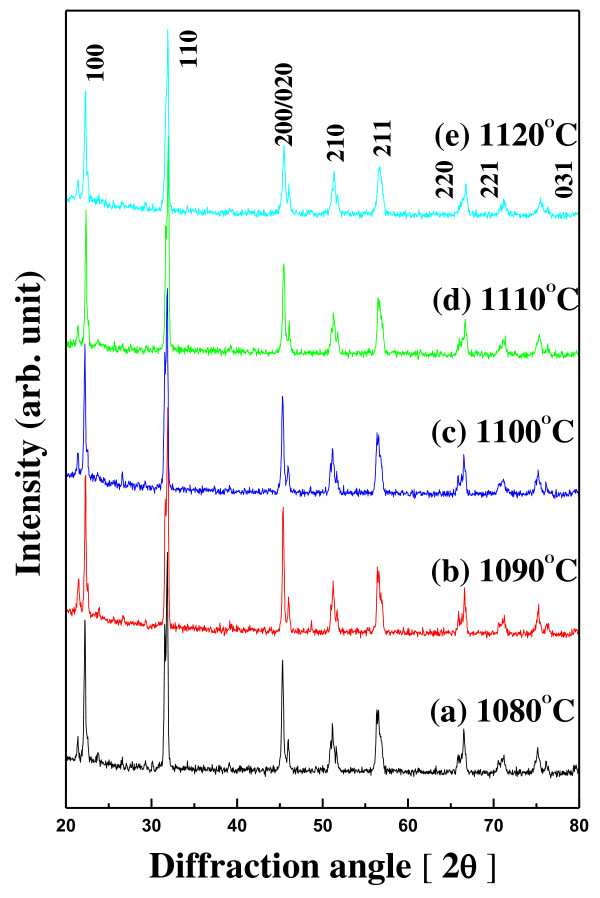
**XRD patterns of 0.98NKN-0.02BZT ceramics**.

**Figure 2:**
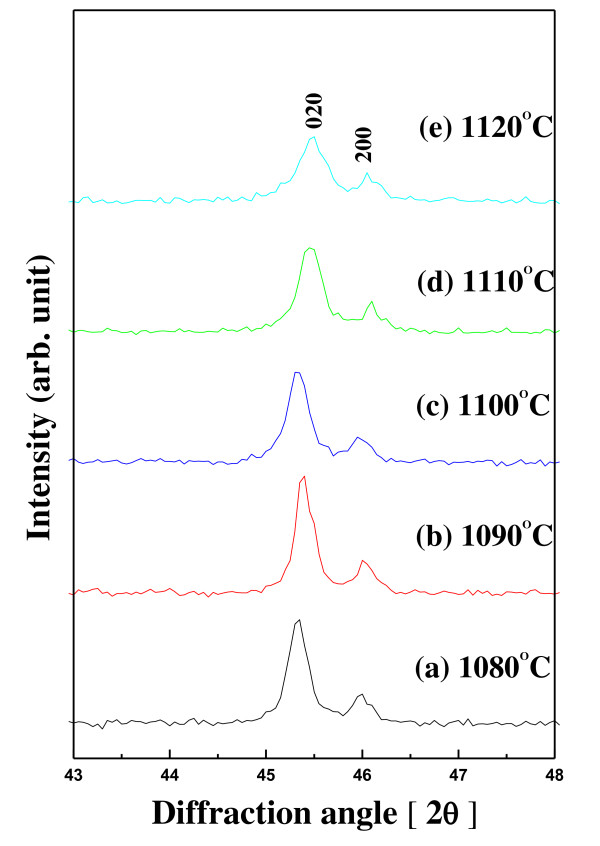
**XRD patterns of 0.98NKN-0.02BZT ceramics near the (020) and (200) planes**.

The XRD patterns of 0.98NKN-0.02BZT ceramics with sintering temperatures were varied from 1,080°C to 1,120°C as shown in Figure 1. As seen from these XRD patterns, the 0.98NKN-0.02BZT phase sintered at various sintering temperatures was well developed without a second phase. It can be seen clearly in Figure 2 that the 0.98NKN-0.02BZT ceramic had an orthorhombic phase that was not changed for all samples. The orthorhombic phases are characterized by (200) and (020) peaks splitting at approximately 45.5°, and when the sintering temperature was increased, the peak form is almost the same. These results indicated that the 0.98NKN-0.02BZT ceramics with various sintering temperatures are regarded to have an orthorhombic structure. However, the degree of crystallization of all samples is completely different. The 0.98NKN-0.02BZT ceramics were well crystallized with increasing sintering temperature. However, as the sintering temperature was increased above 1,090°C, the peak shape became flatter than that of 0.98NKN-0.02BZT ceramics sintered at 1,090°C. It can be inferred that 0.98NKN-0.02BZT ceramics sintered above 1,090°C lost their well-developed orthorhombic structure. These structural results cause a decline of electrical properties such as *d*_33 _and dielectric constant.

**Figure 3:**
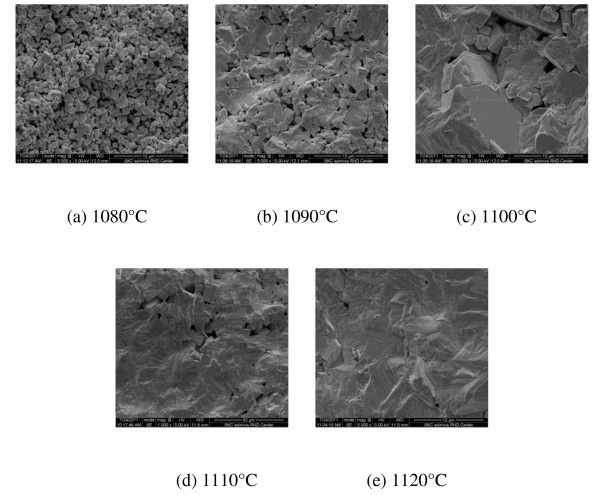
**SEM images of 0.98NKN-0.02BZT ceramics**. (**a**) 1,080°C, (**b**) 1,090°C, (**c**) 1,100°C, (**d**) 1,110°C, and (**e**) 1,120°C.

Figure 3 shows the SEM images of the 0.98NKN-0.02BZT ceramics sintered at various sintering temperatures. As shown in Figure 3a, 0.98NKN-0.02BZT ceramics sintered at 1,080°C showed a small average grain size, although a dense microstructure was formed. It can be inferred that the grain growth was not completed due to low sintering temperature. Figure 2b exhibits the SEM images of 0.98NKN-0.02BZT ceramics sintered at 1,090°C. The cavities have been reduced, and the sample turns into a higher-density microstructure with an increased average grain size. This is according to the kinetic grain growth equation expressed as [13]:

$$\log G=\frac{1}{n}\log t+\frac{1}{n}\left(\log K_{0}-0.434\frac{Q}{RT}\right)$$

where *G *is the average grain size at the time, *n*, the kinetic grain growth exponent, *t*, the sintering time, *K*_0_, a constant, *Q*, the apparent activation energy, *R*, the gas constant, and *T*, the absolute temperature. It can be inferred that increasing sintering temperature improves the grain growth. However, with an increasing sintering temperature above 1,090°C, the microstructure was inhomogeneous and the grain size becomes exceptionally huge. These can be the reason for the deterioration of the relative bulk density over 1,090°C as shown in the SEM images.

**Figure 4:**
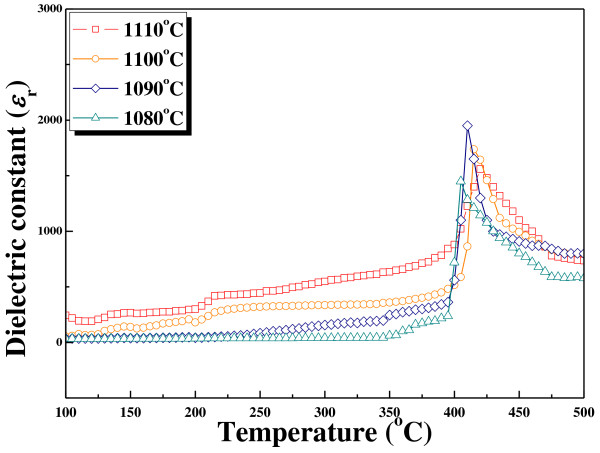
**Temperature-dependent dielectric properties of 0.98NKN-0.02BZT ceramics**.

Figure 4 shows the temperature dependence of the dielectric constant as a function of the temperature for 0.98NKN-0.02BZT ceramics sintered at various sintering temperatures. All samples show transitional peaks and one-transition temperatures at Curie temperature [*T*_c_] of the 0.98NKN-0.02BZT ceramics. The *T*_c _slightly, but not rapidly, decreased with increasing sintering temperature. The *T*_c _for all samples sintered at 1,080°C, 1,090°C, 1,100°C, and 1,110°C is 406°C, 411°C, 417°C, and 421°C, respectively. The *T*_c _increased with increasing sintering temperature owing to considerably increase the *K *ratio in the A-site of NKN ceramics. This phenomenon of gradually increasing *T*_c _for the 0.98NKN-0.02BZT ceramics is similar to that of the NKN system with an increasing *K *ratio. The dielectric constants enhanced with increasing sintering temperature. However, when increasing the sintering temperature above 1,090°C, the dielectric constant decreased. From this decreased dielectric constant, it can be inferred that volatile Na and K ions were evaporated at the high sintering temperature and relative bulk density was decreased. The maximum dielectric constant of 0.98NKN-0.02BZT ceramics is 1,951 sintered at 1,090°C.

**Figure 5:**
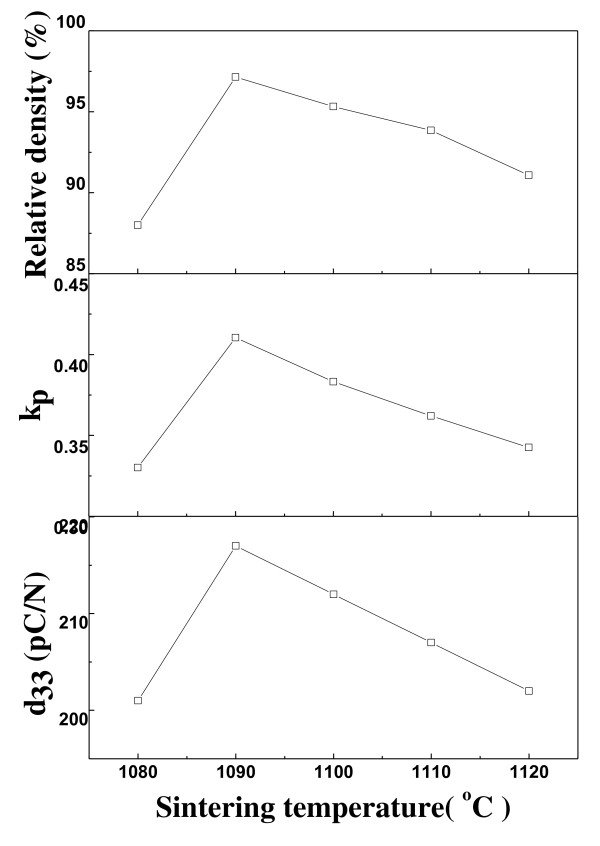
**Piezoelectric properties and relative density of 0.98NKN-0.02BZT ceramics**.

**Figure 6:**
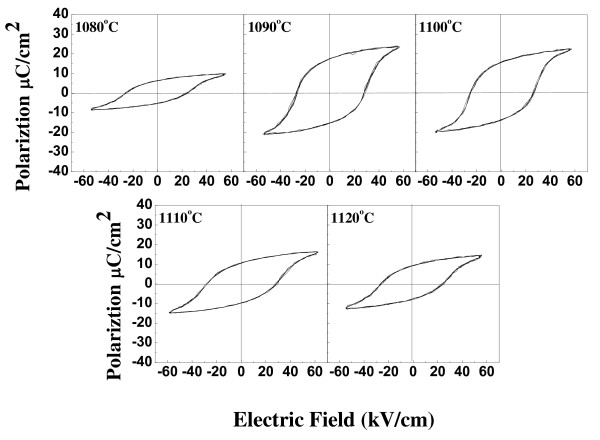
**P-E hysteresis loops of 0.98NKN-0.02BZT ceramics**.

Figure 5 shows the *d*_33_, *k*_p_, and relative density of the poled 0.98NKN-0.02BZT ceramics sintered at various sintering temperatures. It is obvious that *d*_33_, *k*_p_, and relative density have a similar tendency as a function of sintering temperature. The *d*_33_, *k*_p_, and relative density of 0.98NKN-0.02BZT ceramics sintered at 1,080°C are 201 pC/N, 0.33, and 88%, respectively and peaked their maximum values, which are 217 pC/N, 0.41, and 97%, respectively. It can be concluded that the promotion can be attributed to the increase in bulk density, lowering the leakage current, and improving the poling process. With a further increasing sintering temperature above 1,090°C, the piezoelectric properties and relative density decreased. It can be explained that the samples start to heavily volatilize Na and K of 0.98NKN-0.02BZT ceramics. The *k*_p _is calculated by the following equation [14]:

$$\frac{1}{k^{2}}=a\times \frac{f_{\text{r}}}{\left(f_{\text{a}}-f_{\text{r}}\right)}+b$$

where *f*_r _is the resonance frequency, *f*_a _is the antiresonance frequency, *a *= 0.395, and *b *= 0.674 for a planar (*k*_p_) mode. Figure 6 shows the ferroelectric properties of the 0.98NKN-0.02BZT ceramics sintered at various temperatures. The *P*_r _is 5.8 μC/cm^2 ^and the coercive electric field [*E*_c_] is 5.4 kV/cm for 0.98NKN-0.02BZT ceramics sintered at 1,080°C. When the sintering temperature was increased up to 1,090°C, the well-saturated ferroelectric properties were obtained, and the values of *P*_r _and *E*_c _of samples sintered at 1,090°C were 10.3 μC/cm^2 ^and 7.2 kV/cm, respectively. Continuously increasing the sintering temperature above 1,090°C, the ferroelectric properties decreased due to heavy volatilization of Na and K at high sintering temperature. The ferroelectric properties of 0.98NKN-0.02BZT ceramics have a similar tendency as with the piezoelectric and dielectric properties. The increase of ferroelectric properties might be caused by the increase of the relative bulk density that reduces the leakage current, promoting the polarization process.

## Conclusion

In conclusion, the lead-free 0.98NKN-0.02BZT ceramics with a perovskite structure have been sintered at various sintering temperatures. The effects of the sintering temperatures on the structural and electrical properties were investigated. Increasing sintering temperatures improve the grain growth, densification, and electrical properties in effect. However, with an increasing sintering temperature above 1,090°C, the structural and electrical properties have significantly deteriorated. The obtained *d*_33 _is 217 pC/N, which is the highest value in the 0.98NKN-0.02BZT system. The equivalent properties of *T*_c_, *k*_p_, *P*_r_, and dielectric constant values are 411°C, 0.41, 10.3 μC/cm^2^, and 1,951, respectively. Therefore, 0.98NKN-0.02BZT ceramics is a potential candidate for lead-free piezoelectric ceramics.

## Competing interests

The authors declare that they have no competing interests.

## Authors' contributions

S-HL carried out the experiments which show the electrical properties and drafted the manuscript. S-GL carried out the experiments which show the structural properties and reviewed the manuscript. Y-HL participated in the design of this study and reviewed the manuscript finally. All authors read and approved the final manuscript.

## References

[B1] Hansen P Hennings D Schreinemacher H High-K dielectric ceramics from donor/acceptor-Co doped (Ba_1-x_Ca_x_)(Ti_1-y_Zr_y_)O_3_ J Am Ceram Soc 1998 81 1369

[B2] Lee SH Lee YH Piezoelectric and dielectric properties of (Na_0.44_K_0.52_)Nb_0.84_O_3_-Li_0.04_(Sb_0.06_Ta_0.1_)O_3 _ceramics with sintering temperature Electronic Materials Letters 2011 7 205 10.1007/s13391-011-0905-1

[B3] Nam SP Lee SG Bae SG Lee YH Electrical properties of (Bi,Y)_4_Ti_3_O_12 _thin films grown by RF sputtering method J Electrical Engineering & Technology 2007 2 9822216090

[B4] Noh HJ Lee SG Nam SP Dielectric and pyroelectric properties of Dy-doped BSCT thick films by screen-printed method J Electrical Engineering & Technology 2009 4 52722216090

[B5] Cho IJ Yun KS Nam HJ A high-speed single crystal silicon AFM probe integrated with PZT actuator for high-speed imaging applications J Electrical Engineering & Technology 2011 6 11922216090

[B6] Matsubara M Yamaguchi T Kikuta K Hirano S Sinterability and piezoelectric properties of (K,Na)NbO_3 _ceramics with novel sintering aid Jpn J Appl Phys 2004 43 7159 10.1143/JJAP.43.7159

[B7] Hollenstein E Davis M Damjanovic D Setter N Piezoelectric properties of Li- and Ta- modified (K_0.5_Na_0.5_)NbO_3 _ceramics Appl Phys Lett 2005 87 182905 10.1063/1.2123387

[B8] Zhang SJ Xia R Shrout TR Zang GZ Wang JF Piezoelectric properties in perovskite 0.948(K_0.5_Na_0.5_)NbO_3_-0.052LiSbO_3 _lead-free ceramics J Appl Phys 2006 100 104108 10.1063/1.2382348

[B9] Bae HJ J K Hong JP Dielectric properties of Ti-doped K(Ta,Nb)O_3 _thin films for tunable microwave applications J Electrical Engineering & Technology 2006 1 12022216090

[B10] Yuan GL Or SW Enhanced piezoelectric and pyroelectric effects in single-phase multiferroic Bi_1-x_Nd_x_FeO_3 _(x = 0-0.15) ceramics Appl Phys Lett 2006 88 062905 10.1063/1.2169905

[B11] Kim MS Jeon YM IM YM Lee YH Nam TH Crystallization behavior of Ti-(50-x)Ni-xCu(at%) (x = 20-30) alloy ribbons Trans Electr Electron Mater 2011 12 20 10.4313/TEEM.2011.12.1.20

[B12] Lee SH Electromagnetic properties of Bi system J Electrical Engineering & Technology 2007 2 10222216090

[B13] Chen TY Chu SY Juang YD Effects of sintering temperature on the dielectric and piezoelectric properties of Sr additive Sm-modified PbTiO_3 _ceramics Sens Actuator A Phys 2002 102 6 10.1016/S0924-4247(02)00382-5

[B14] Matsubara M Yamaguchi T Kikata K Hirano S Effect of Li substitution on the piezoelectric properties of potassium sodium niobate ceramics Jpn J Appl Phys 2005 44 6136 10.1143/JJAP.44.6136

